# Dear Doctor Letters regarding citalopram and escitalopram: guidelines vs real-world data

**DOI:** 10.1007/s00406-022-01392-x

**Published:** 2022-02-25

**Authors:** Mateo de Bardeci, Waldemar Greil, Hans Stassen, Jamila Willms, Ursula Köberle, René Bridler, Gregor Hasler, Siegfried Kasper, Eckart Rüther, Stefan Bleich, Sermin Toto, Renate Grohmann, Johanna Seifert

**Affiliations:** 1 Department of Psychiatry and Psychotherapy, Ludwig Maximilian University, Nussbaumstr. 7, 80331 Munich, Germany; 2 Psychiatric Private Hospital, Sanatorium Kilchberg, Kilchberg-Zurich, Switzerland; 3 Institute for Response-Genetics, Psychiatric University Hospital (KPPP), Zurich, Switzerland; 4 Arzneimittelkommission der Deutschen Ärzteschaft, Berlin, Germany; 5 Psychiatry Research Unit, University of Fribourg, Fribourg, Switzerland; 6 Department of Molecular Neuroscience, Medical University of Vienna, Vienna, Austria; 7 Department of Psychiatry, Social Psychiatry and Psychotherapy, Hannover Medical School, Hannover, Germany

**Keywords:** Dear Doctor Letter, DDL, Direct Healthcare Professional Communications, DHPCs, Citalopram, QTc prolongation, Torsade de Pointes, TdP

## Abstract

Dear Doctor Letters (DDLs, Direct Healthcare Professional Communications) from 2011 provided guidance regarding QTc-prolonging effects with risk of torsade de pointes during treatment with citalopram and escitalopram. This study examines the DDLs’ effects on prescription behavior. Data from 8842 inpatients treated with citalopram or escitalopram with a primary diagnosis of major depressive disorder (MDD) were derived from a European pharmacovigilance study (Arzneimittelsicherheit in der Psychiatrie, AMSP) from 2001 to 2017. It was examined to what extent new maximum doses were adhered to and newly contraindicated combinations with QTc-prolonging drugs were avoided. In addition, the prescriptions of psychotropic drugs before and after DDLs were compared in all 43,480 inpatients with MDD in the data set. The proportion of patients dosed above the new limit decreased from 8 to 1% in patients ≤ 65 years and from 46 to 23% in patients > 65 years old for citalopram versus 14–5% and 47–31% for escitalopram. Combinations of es-/citalopram with other QTc-prolonging psychotropic drugs reduced only insignificantly (from 35.9 to 30.9%). However, the proportion of patients with doses of quetiapine > 150 mg/day substantially decreased within the combinations of quetiapine and es-/citalopram (from 53 to 35%). After the DDLs, prescription of citalopram decreased and of sertraline increased. The DDLs’ recommendations were not entirely adhered to, particularly in the elderly and concerning combination treatments. This might partly be due to therapeutic requirements of the included population. Official warnings should consider clinical needs.

**Supplementary Information:**

The online version contains supplementary material available at 10.1007/s00406-022-01392-x.

## Introduction

Dear Doctor Letters (DDLs), also known as Direct Healthcare Professional Communications (DHPCs), are paper-based warning letters with the intention of informing physicians about newly detected drug risks and recommendations to reduce these risks [1]. However, the effectiveness of DDLs has been challenged [2–5], in part due to insufficient quality of warning instructions in DDLs [6]. Furthermore, a Dutch study found that DDLs regarding the risk of hospitalization are less effective than DDLs related to the risk of death or disability [7]. In addition, advisories recommending closer monitoring of patients appear not to have a large and sustained impact on clinical practice [8]. Since prescription behavior is influenced by several factors and not only by DDLs, it is difficult to attribute potential changes in prescription behavior directly to specific warning messages [9].

In 2011, DDLs (called “Rote Hand-Briefe”, RHB, in Germany) regarding citalopram and escitalopram were sent out by Lundbeck in the three German-speaking countries (Germany, Austria and Switzerland; [10, 11]). Citalopram and its active S-isomer escitalopram are selective serotonin reuptake inhibitors (SSRIs) and are commonly used in the treatment of major depressive disorder (MDD) [12]. Due to their efficacy and tolerability, they are widely used in adult as well as in geriatric populations [13].

The DDLs recommended a reduction of the maximum daily dose of citalopram and escitalopram due to the risk of QTc prolongation. In addition, the DDLs advised against the combination of es-/citalopram with other potentially QTc-prolonging drugs. Similar instructions were issued by the US Food and Drug Administration (FDA) [14] as well as by the European Medicines Agency (EMA) [15]. The FDA further recommended electrocardiogram (ECG) monitoring in patients taking citalopram who are at particular risk of QTc prolongation [14] such as older patients, females, as well as patients with a history of heart disease, hypokalemia or hypomagnesemia [16, 17].

The warnings were based on a potential dose-dependent QTc prolongation during treatment with es-/citalopram with risk of Torsade de Pointes (TdP), a polymorphic ventricular tachycardia that can lead to sudden cardiac death. Compared to placebo, maximum mean prolongations in the individually corrected QT intervals were 8.5 and 18.5 ms (ms) for 20 mg/day and 60 mg/day citalopram, respectively [14]. Prolongation of the corrected QTc interval was estimated to be 12.6 ms for citalopram 40 mg/day [14]. For escitalopram, the mean prolongation in the individually corrected QTc intervals was 4.3 ms and 10.7 ms for 10 mg/day and 30 mg/day, respectively, compared to placebo [11]. Moreover, the FDA received post-marketing reports of QTc interval prolongation and TdP associated with citalopram which further substantiated the reduction of the maximum daily dose of citalopram [14].

Because of the observed dose-dependent QTc interval prolongation with risk of TdP, the DDLs in 2011 recommended new maximum doses: Citalopram should no longer be prescribed at doses > 40 mg/day in patients ≤ 65 years and > 20 mg/day in patients > 65 years [10]. The corresponding dosages of escitalopram are 20 mg/day and 10 mg/day, respectively [11].

The reduction of the maximum daily dose of es-/citalopram led to much controversy since dosages as high as 30-60 mg/day of citalopram and 20 mg/day of escitalopram may be needed to achieve full clinical response in MDD, even in older patients [13, 18, 19]. The reduction of prescribed dosages of citalopram to a new safety limit of 40 mg/day has been associated with a significant increase in all-cause and depression-related hospitalizations observed in a population of 35,848 veterans consisting predominantly of men [20]. Thus, physicians are faced with the difficult decision of complying with the DDL recommendations to minimize potential risk of QTc interval prolongation while risking potential destabilization of mental health as a result of dose reduction [13] or of discontinuing useful combinations.

The warning not to combine es-/citalopram with other potentially QTc-prolonging drugs was particularly controversial, since the combined use of SSRIs with antipsychotic drugs (APDs), many of which may lead to a prolongation of the QTc interval, especially quetiapine [17], is a common practice in the treatment of patients suffering from MDD [21].

The German Association for Psychiatry, Psychotherapy and Psychosomatics (Deutsche Gesellschaft für Psychiatrie und Psychotherapie, Psychosomatik und Nervenheilkunde e.V., DGPPN) published a statement that the warnings in the DDLs were overdrawn. It has been postulated that the restricted use of es-/citalopram would limit clinical treatment options [22]. A similar statement was made by the corresponding Austrian association [23] based on data of the drug safety project “Arzneimittelsicherheit in der Psychiatrie” (AMSP) about cardiovascular adverse drug reactions (ADR) related to antidepressant drugs (ADDs) which was published in 2015 [24]. The AMSP data showed that SSRIs in general, including es-/citalopram, have a low cardiovascular risk profile. Not a single case of QTc prolongation was found for es-/citalopram when imputed alone for this ADR in the large AMSP dataset from Germany, Austria and Switzerland between 1993 and 2010. Citalopram, like escitalopram, was imputed for QTc interval prolongation in combination with APDs in two cases. The authors of the Austrian professional society [23] concluded that the EMA’s instructions [15] regarding dose reduction of es-/citalopram, contraindication and co-medication should be regarded as inappropriate in practice and in need of revision [23]. Specifically, they emphasized that the cardiac risk of escitalopram is relevantly lower than of citalopram. McKean et al. [25] questioned whether the FDA warnings on es-/citalopram have done more harm than good.

This study examines how the DDLs have affected real-life prescribing behavior in the treatment of MDD of psychiatric inpatients. To what extent were the new maximum dosages of es-/citalopram adhered to and combinations with other potentially QTc-prolonging drugs—in particular quetiapine—avoided? Furthermore, it will be examined whether sex and age influenced the prescribing behavior recommended by the DDLs, since females and older patients are particularly vulnerable to cardiac ADRs. In addition, potential long-lasting general changes in the prescribing behavior of psychotropic drugs after the DDLs were identified (“carry-over effects”).

## Materials and methods

### Data source

The prescription data analyzed in the present study were gathered by AMSP. AMSP is an ongoing European multi-center drug safety program which has been collecting data on psychopharmacotherapy and severe ADRs from psychiatric hospitals in a naturalistic setting since 1993. AMSP’s pharmacovigilance methods have been described in detail previously [26, 27]. Briefly, AMSP consists of two principal data collections (prescription data and severe ADRs) from a total of 116 hospitals in Germany, Switzerland and Austria, as well as temporarily from one hospital each in Belgium and Hungary. The number of participating hospitals increased from nine in 1994 to 52 in 2017 [21]. In a cross-sectional approach, the participating hospitals record drug prescriptions for all inpatients under surveillance on two reference days per year. All drugs (including dosage for psychotropic drugs) administered on these days are assessed along with the patients’ age, sex and psychiatric diagnoses. The current evaluation includes data from reference day surveys from the years 2001 to 2017.

Evaluations of the AMSP database have been approved by the Ethics Committee of the University of Munich and the Ethics Committee of the Hannover Medical School (Nr. 8100 BO S 2018). This study adheres to the Declaration of Helsinki and its later amendments. The AMSP program is a continuous observational post-marketing drug surveillance program and does not interfere with the ongoing clinical treatment of patients under surveillance.

### Study population

8841 patients between ≥ 18 and < 90 years of age, hospitalized between 2001 and 2017, with a primary diagnosis of MDD and at least one prescription of citalopram or escitalopram were investigated. In the total AMSP study population consisting of 43,480 inpatients with MDD, we additionally compared the general changes in prescribing behavior of psychotropic medication before and after DDL in 2011 (see Supplementary Data; see also Seifert et al. [21]).

### Statistical analysis

Data were analyzed in an explorative approach. We studied the time periods 2005–2010 (T1) and 2012–2017 (T2), i.e., 6 years before and after DDLs. We divided the study population by sex and age (≤ 65 and > 65 years of age), as we computed the total number of patients prescribed es-/citalopram, number of patients with dosages above the DDL limit and number of patients with combinations of APDs. To assess the impact of the DDLs, we calculated the risk ratio (RR) between T1 and T2 as well as the respective 95% confidence interval (CI). We consider a RR statistically significant if the CI does not include 1. The results of the descriptive statistics are presented as two separate tables for citalopram and escitalopram.

Furthermore, we present the evolution of combinations of es-/citalopram with potentially QTc-prolonging psychotropic drugs (“risky drugs”) over time from 2001 to 2017. Along with the *p* value from the t-test, we calculated the standard error of the mean.

To determine the set of potentially QTc-prolonging psychotropic drugs, we extracted data from the review by Wenzel-Seifert et al. [17]. A drug is considered “risky” if at least one of the following criteria is fulfilled according to Wenzel-Seifert et al. [17]: generally accepted elevated risk of TdP, severe QTc prolongation (≥ 17 ms), moderate QTc prolongation (≥ 9 and < 16 ms), and at least rare case reports of TdP (for details see Supplementary Material, Table 1).

**Table 1:** Study population summary

Total number of patients	8841	–	–
Number of males	3173	–	35.9%
Number of females	5668	–	64.1%
Average age of males (in years)	48.0	sd	15.8
Average age of females (in years)	50.0	sd	17.2
Mild MDD (“F32.0”, “F33.0”)	120	–	1.4%
Males	44	–	1.4%
Females	76	–	1.3%
Moderate MDD (“F32.1”, “F33.1”)	2587	–	29.3%
Males	886	–	27.9%
Females	1701	–	30.0%
Severe MDD (“F32.2”, “F33.2”)	5005	–	56.6%
Males	1814	–	57.2%
Females	3191	–	56.3%
Severe MDD with psychosis (“F32.3”, “F33.3”)	913	-	10.3%
Males	346	-	10.9%
Females	567	-	10.0%
Other or no info MDD (“F32”, “F32.8”, “F32.9”, “F33”, “F33.4”, “F33.8”, “F33.9”)	216	-	2.4%
Males	83	-	2.6%
Females	133	-	2.4%

*sd* standard deviation

**Fig. 1:**
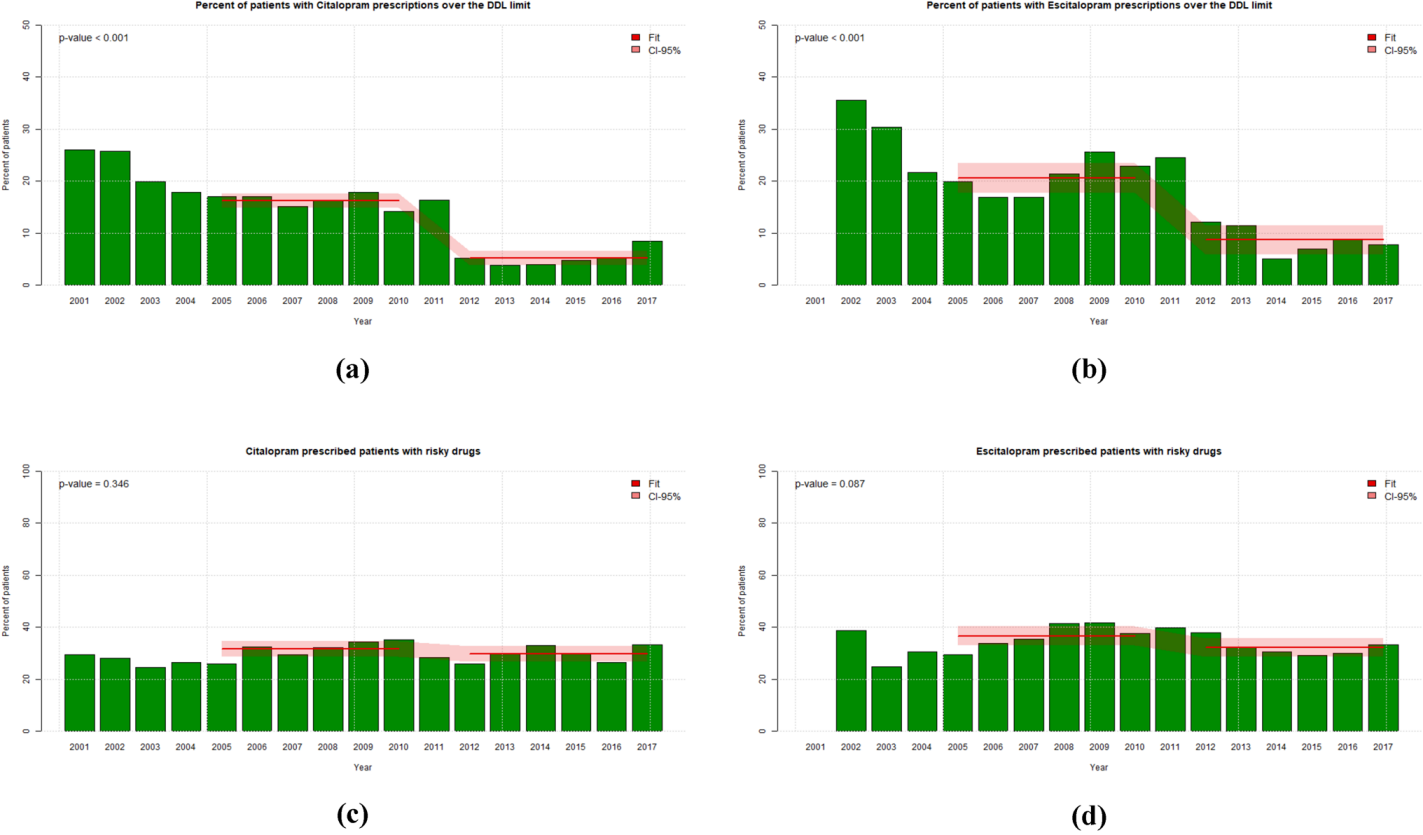
Citalopram and escitalopram: dosages and combinations: comparison 6 years before and after DDL (in 2011). **a** Percent of patients with citalopram prescriptions above the DDL dose limit. Statistics 2005–2010 vs 2012–2017: 16.2% vs 5.2%, *p* < 0.001 (for comparison: 2008–2010 vs 2012–2014: 16.1% vs 4.3%, *p* < 0.001). **b** Percent of patients with escitalopram prescriptions above the DDL dose limit. Statistics 2005–2010 vs 2012–2017: 20.6% vs 8.7%; *p* < 0.001 (for comparison: 2008–2010 vs 2012–2014: 23.3% vs 9.5%, *p* = 0.006). **c** Combinations of citalopram with potentially QTc-prolonging drugs (in percent). Statistics 2005–2010 vs 2012–2017: 31.5% vs 30.0%, *p* > 0.05. **d** Combinations of escitalopram with potentially QTc-prolonging drugs (in percent). Statistics 2005–2010 vs 2012–2017: 36.5% vs 32.1%, *p* > 0.05. *p* values according *t*-tests. The fits of the mean refer to 6 years before and after the DDLs (in 2011)

In addition, we calculated short-term effects of the DDLs by comparing prescription trends 3 years before and 3 years after the DDLs, i.e., 2008–2010 versus 2012–2014 (see legends of Fig. 1 and Supplement).

## Results

The sample (MDD patients treated with citalopram or escitalopram) consists of 3173 males (35.9%) and 5668 females (64.1%). Table 1 shows a summary of the sample composition. Most patients suffered from severe depression; 81% were ≤ 65 and 19% > 65 years old.

For the time period T1 (2005–2010) and T2 (2012–2017), the number of patients with prescriptions above the DDL limits, as well as the combinations with APDs are shown in Table 2 (citalopram) and in Table 3 (escitalopram). The results are presented separately for different patient groups: young (≤ 65 years of age), elderly (> 65 years of age), male, and female. The RR comparing T1 with T2 are presented in the tables as well, along with its 95% CI.

**Table 2:** Prescriptions of citalopram before and after DDL

Citalopram	T1 + T2		T1 (2005–2010)		T2 (2012–2017)		T1 vs. T2
	*N*	%	*N*	%	*N*	%	RR (95% CI)
Females and males							
All ages	2890	100	1490	100	1400	100	–
Above the DDL limit	311	10.8	241	16.2	70	5.0	0.60 (0.46–0.78)*
+ APD	1253	43.4	725	48.7	528	37.7	0.90 (0.82–0.97)*
Young (≤ 65 years)	2319	100	1154	100	1165	100	–
> 40 mg/day	104	4.5	88	7.6	16	1.4	0.47 (0.28–0.80)*
+ APD	971	41.9	543	47.1	428	36.7	0.90 (0.82–0.99)*
Elderly (> 65 years)	571	100	336	100	235	100	–
> 20 mg/day	207	36.3	153	45.5	54	23.0	0.74 (0.57–0.97)*
+ APD	282	49.4	182	54.2	100	42.6	0.90 (0.75–1.08)
Females							–
All ages	1848	100	959	100	889	100	
Above the DDL limit	208	11.3	163	17.0	45	5.1	0.59 (0.43–0.81)*
+ APD	807	43.7	466	48.6	341	38.4	0.90 (0.81–1.00)
Young (≤ 65 years)	1,435	100	708	100	727	100	-
> 40 mg/day	57	4.0	50	7.1	7	1.0	0.42 (0.19–0.92)*
+ APD	607	42.3	334	47.2	273	37.6	0.91 (0.80–1.02)
Elderly (> 65 years)	413	100	251	100	162	100	–
> 20 mg/day	151	36.6	113	45.0	38	23.5	0.75 (0.55–1.03)
+ APD	200	48.4	132	52.6	68	42.0	0.91 (0.73–1.13)
Males							–
All ages	1,042	100	531	100	511	100	
Above the DDL limit	103	9.9	78	14.7	25	4.9	0.62 (0.40–0.96)*
+ APD	446	42.8	259	48.8	187	36.6	0.88 (0.76–1.02)
Young (≤ 65 years)	884	100	446	100	438	100	–
> 40 mg/day	47	5.3	38	8.5	9	2.1	0.54 (0.26–1.10)
+ APD	364	41.2	209	46.9	155	35.4	0.89 (0.75–1.04)
Elderly (> 65 years)	158	100	85	100	73	100	–
> 20 mg/day	56	35.4	40	47.1	16	21.9	0.72 (0.44–1.17)
+ APD	82	51.9	50	58.8	32	43.8	0.88 (0.64–1.21)

*APD* antipsychotic drug, *DDL* Dear Doctor Letters, *T1* time period from 2005 to 2010, *T2* time period from 2012 to 2017. Above the DDL limit = the DDL recommendation states that citalopram should no longer be used at doses above 40 mg/day in patients ≤ 65 years and above 20 mg/day in patients > 65 years. *RR* risk ratio

*Statistically significant at a significance level of 5% (95% confidence interval, in which 1 is not included

**Table 3:** Prescriptions of escitalopram before and after DDL

Escitalopram	T1 + T2		T1 (2005–2010)		T2 (2012–2017)		T1 vs. T2
	*N*	%	*N*	%	*N*	%	RR (95% CI)
Females and males							
All ages	3906	100	1820	100	2086	100	–
Above the DDL limit	558	14.3	378	20.8	180	8.6	0.68 (0.58–0.81)*
+ APD	1859	47.6	909	49.9	950	45.5	0.96 (0.90–1.03)
Young (≤ 65 years)	3207	100	1443	100	1764	100	–
> 20 mg/day	283	8.8	202	14.0	81	4.6	0.62 (0.48–0.79)*
+ APD	1463	45.6	702	48.6	761	43.1	0.95 (0.88–1.02)
Elderly (> 65 years)	699	100	377	100	322	100	–
> 10 mg/day	275	39.3	176	46.7	99	30.7	0.83 (0.69–1.01)
+ APD	396	56.7	207	54.9	189	58.7	1.03 (0.90–1.17)
Females							
All ages	2503	100	1159	100	1344	100	–
Above the DDL limit	349	13.9	239	20.6	110	8.2	0.67 (0.54–0.83)*
+ APD	1161	46.4	570	49.2	591	44.0	0.95 (0.88–1.04)
Young (≤ 65 years)	2007	100	891	100	1116	100	–
> 20 mg/day	167	8.3	123	13.8	44	3.9	0.58 (0.42–0.81)*
+ APD	879	43.8	425	47.7	454	40.7	0.93 (0.85–1.03)
Elderly (> 65 years)	496	100	268	100	228	100	–
> 10 mg/day	182	36.7	116	43.3	66	28.9	0.84 (0.66–1.07)
+ APD	282	56.9	145	54.1	137	60.1	1.05 (0.90–1.22)
Males							
All ages	1403	100	661	100	742	100	–
Above the DDL limit	209	14.9	139	21.0	70	9.4	0.71 (0.54–0.92)*
+ APD	698	49.8	339	51.3	359	48.4	0.98 (0.88–1.08)
Young (≤ 65 years)	1200	100	552	100	648	100	–
> 20 mg/day	116	9.7	79	14.3	37	5.7	0.67 (0.46–0.97)*
+ APD	584	48.7	277	50.2	307	47.4	0.98 (0.87–1.10)
Elderly (> 65 years)	203	100	109	100	94	100	–
> 10 mg/day	93	45.8	60	55.0	33	35.1	0.82 (0.60–1.14)
+ APD	114	56.2	62	56.9	52	55.3	0.99 (0.77–1.26)

*APD *antipsychotic drug, *DDL *Dear Doctor Letters, *T1 *time period from 2005 to 2010, *T2* time period from 2012 to 2017. Above the DDL limit = the DDL recommendation states that escitalopram should no longer be used at doses above 20 mg/day in patients ≤ 65 years and above 10 mg/day in patients > 65 years. *RR* risk ratio

*Statistically significant at a significance level of 5% (95% confidence interval in which 1 is not included)

Overall, we found a statistically significant reduction of doses above the DDL limit (RR = 0.60) for citalopram considering all ages (Table 2, Fig. 1a). During T1, 16.2% of all patients were dosed above the DDL limit, whereas during T2 this applied to only 5% of patients. The reduction of doses above the DDL limit is more pronounced in young patients (RR = 0.47) than in the elderly (RR = 0.74). In addition, we found a significant reduction of combinations with APDs (RR = 0.90), similar for both age groups. During T1, 48.7% of the patients received citalopram in combination with an APD, whereas this was the case in 37.7% of patients in T2 (see also Fig. 5 Suppl.).

We did not find evidence of a significant reduction or increase of combinations of escitalopram with APDs (T1 49.9%, T2 45.5%; Table 3; see also Fig. 6 Suppl.). Further, we found a reduction of dosages above the DDL limits for patients considering all ages (RR = 0.68), but the reduction is slightly less pronounced than in the case of citalopram (Fig. 1b). The dose reduction of escitalopram (T1 20.8% and T2 8.6% across all ages) was observed especially among young patients (T1 14.0% and T2 4.6%); the respective numbers for the elderly were 46.7% and 30.7% (Table 3).

The evolution of patients treated with combinations of citalopram or escitalopram with any potentially QTc-prolonging psychotropic drug (“risky drugs”) from 2001 to 2017 is shown in Fig. 1c, d. The mean values during 2005–2010 and 2012–2017 (depicted as a red line) were 31.5% and 30.0% for citalopram, and 36.5% and 32.1% for escitalopram, respectively (*p* > 0.05, not significant).

We did not find substantial differences between sexes regarding dosages and combinations of citalopram as well as of escitalopram.

The evaluation of prescription data before and after the DDL shows that the general trends in the treatment of MDD with psychotropic medication remained unchanged (data shown in Supplementary Material): a decrease in the use of benzodiazepines and hypnotic drugs over time, with essentially the same frequency of prescription of ADDs and APDs. The two most prescribed APDs (i.e., quetiapine and olanzapine [see also [21] and their combined use with es-/citalopram remained unchanged during the time period of this analysis. Among ADDs, however, there were changes: citalopram was used less, whereas sertraline was used more often after the DDLs. Use of escitalopram initially decreased after the DDLs, then increased again. The proportion of patients taking more than 150 mg/day of quetiapine, another substance with potentially QTc-prolonging properties, decreased from 52.7 to 36.4% when given in combination with es-/citalopram. Only a slight reduction of concomitantly used psychotropic drugs was seen when comparing 6 years before and after DDL: from an average of 2.7–2.5 drugs (*p* < 0.001, see Supplementary Data).

## Discussion

To our knowledge, this is the only study that describes the impact of the DDLs regarding citalopram and escitalopram in the treatment of MDD in hospitalized patients that (1) presents data over a long period of time (up to 6 years after DDLs), (2) examines the influence of the DDL warning on dosages as well as on combinations with other drugs, (3) considers both the influence of age and sex, and (4) determines the impacts of the DDLs on the prescription behavior of psychotropic medication in general as possible indicators for spill-over effects.

The results show that following the DDLs in 2011, a significant reduction in doses of citalopram and escitalopram above the DDL limit in patients across all ages was observed. When comparing the 6 years before and after the DDL, the proportion of patients with dosages above the DDL limit decreased significantly from 16 to 5% (citalopram) and 21–9% (escitalopram). In the younger age group, there were only a few “overdosed” patients even before the DDL, as doses of more than 40 mg of citalopram or 20 mg of escitalopram were rarely used. The proportion of “overdosed” patients in this age group fell from 8 to 1% (citalopram) and 14–5% (escitalopram). Among the elderly, on the other hand, a higher percentage of patients had previously been treated with doses higher than 20 mg and 10 mg, i.e., 46% (citalopram) and 47% (escitalopram). Even after the DDLs, the risk of treatment with these elevated doses remained relatively high among elderly patients with 23% (citalopram) and 31% (escitalopram). It is noteworthy that sex did not have a detectable influence on drug dose although women have a higher risk of QTc prolongation [17]. It is important to note, that these results remain consistent when comparing short-term effects of only 3 years before and after the DDLs.

Overall, the recommendations in the DDLs were followed only to a limited extent. DDL dosage limits were adhered to more frequently in younger than in older patients although the latter are at greater risk of developing QTc prolongation and TdP [28]. The need for doses > 20 mg citalopram or > 10 mg escitalopram in the elderly seems to be high. Similarly, among Canadian outpatients, the reduction in “overdoses” of citalopram according to DDL was found to be more pronounced in younger than in older people: from 15 to 5% in patients < 65 years of age and 28–19% in the elderly [29].

A more recent study relativizes the DDLs’ warnings. In a real-world geriatric setting including 137 patients, no association was found between es-/citalopram and QTc prolongation, nor was there a case of TdP. Age was found to be a relevant independent risk factor for QTc prolongation. However, the maximum doses in the DDL are not supported by the study [13]. The conclusion drawn from this study is questionable given the rarity of QTc prolongation and of TdP as an ADR of es-/citalopram and the small number of cases, a fact that the authors themselves point out in their limitations.

Compared to the dose limit, the DDLs’ recommendation to avoid combinations of es-/citalopram with other potentially QTc-prolonging drugs appears even more difficult to comply with. Regarding the combinations with APDs, there was only a small decrease from 49 to 38% for citalopram and a not significant reduction of 50–46% for escitalopram (all ages), again with sex not playing a notable role. Among the elderly, there were statistically insignificant changes in the use of the formally contraindicated combinations with APDs from 54 to 43% (citalopram) and from 55 to 59% (escitalopram). For all ages, the combinations with “risky drugs”, i.e., psychotropic drugs with a clear risk for QTc prolongation or TdP remained high at about 35% of all patients.

The high proportion of patients treated with potentially QTc-prolonging psychotropic drug combinations might at least partly be explained by the lack of familiarity of physicians in regard to QTc-prolonging properties of the different drugs. Furthermore, the warnings do not provide a specific overview of potentially QTc-prolonging drugs which explicitly should not be co-prescribed with es-/citalopram.

Combinations, especially with sedating APDs, e.g., quetiapine (one of the “risky drugs”), are particularly helpful at the beginning of treatment and might be useful in managing or avoiding the onset of suicidal ADRs [30].

As suggested by the FDA, one possible measure to reduce the risk of TdP is regular ECGs during treatment with es-/citalopram. After this recommendation had been made, it was reported that patients returned to prewarning levels of ECG monitoring within months in all age groups. Lack of responsiveness to the FDA warnings may be due to many factors, including lack of clarity about which individuals should undergo ECG monitoring and how often [31]. In an evaluation of 6,670 inpatients treated with ADDs in a tertiary care hospital (with all medical departments) in Switzerland, co-administration of explicitly contraindicated QTc-prolonging drugs were used in the treatment of 52.0% and 49.9% of all users of citalopram and escitalopram, respectively, but ECG monitoring was documented in 17.3% of these cases only [32].

In addition, in 872 patients under treatment with second-generation antipsychotic (SGA), combinations that were formally contraindicated were frequently prescribed (in 112 hospitalisations). ECGs were performed in less than half of the cases and clinically relevant cardiac ADRs were detected only in two cases (QTc prolongation). According to the authors, cost and benefit of ECG monitoring should be considered [33]. However, due to the low costs and high availability, clinicians should urgently consider ECG monitoring in all patients treated with potentially QTc-prolonging combinations. The frequency of ECG controls is still subject to discussion.

Another measure to reduce the risk of critical QTc prolongation is the reduction of concomitantly used psychotropic drugs. The only slight decrease of concomitantly administered psychotropic drugs (see Supplementary Data) demonstrates that this particular aspect is very difficult to adhere to within the treatment of psychiatric inpatients.

Comparing prescription data before and after the DDL shows that some of the general trends in the treatment of MDD remained unchanged, e.g., the desirable decline in the prescription of tranquillizing drugs. On the other hand, the preferred use of sertraline is evidence-based, as sertraline has a much lower risk of TdP than citalopram (sertraline = TdP 3 risk, citalopram = TdP 1 risk, [28]). As a further potentially spill-over effect of the DDLs, the proportion of patients taking high doses of quetiapine—the “risky drug” most often combined with es-/citalopram—above 150 mg (across all ages) in combination with es-/citalopram decreased from 53 to 36% of patients. On the other hand, the percentage of patients who received low doses of quetiapine (≤ 50 mg/day) in the combination of quetiapine with es-/citalopram increased strongly (from 22 to 42%). This may indicate that quetiapine was primarily used due to its sedative and sleep-inducing effects rather than its antidepressant effect.

Previous studies have evaluated the risk of quetiapine-induced QTc prolongation in relation to dose and come to inconsistent conclusions. A review of 12 case reports of quetiapine-induced QTc prolongation including five cases of quetiapine overdose reported that the dose of quetiapine did not significantly affect the risk of QTc prolongation. When given at a therapeutic dose, QTc prolongation was observed at daily dosages between 25 and 800 mg [34]. In a prospective cohort analysis of critically ill patients in intensive care similarly concluded, that dose and the QTc prolongation were irrespective of one another. The only significant determinant of quetiapine-induced QTc prolongation was the concomitant use of other potentially QTc-prolonging drugs [35]. A South Korean study suggests that similar to es-/citalopram, that quetiapine-induced QTc prolongation shows a dose–response relationship in a population of healthy volunteers [36]. The influence of dosing of quetiapine and es-/citalopram when used concomitantly and the risk of QTc prolongation requires further investigation.

The results of the present study show that the instructions in the DDLs were not comprehensively implemented. However, the practice corresponded to a certain extent to the recommendations of the professional societies, which described the DDL recommendations as ‘exaggerated’ and ‘impracticable’ [22, 23]. For example, the maximum doses of citalopram and escitalopram were largely adhered to in younger patients, but less so among older ones. The often useful combinations with APDs were still given in many cases, even if they were now contraindicated. However, the prescription data presented in this analysis without information on the occurrence of the ADRs in question (i.e., QTc prolongation and TdP) cannot clarify to what extent a deviation from the warnings of the DDLs is possible without endangering the patients. Our findings demonstrate that the treating physicians seem to value the possible benefits more highly than the possible harm from the combinations of es-/citalopram with QTc-prolonging drugs and from high dosages of es-/citalopram in the elderly. Several AMSP evaluations of drug use data have revealed similar discrepancies between official guidelines and clinical practice, especially evaluations on bipolar depression [37] and borderline personality disorder [38] as well as dosage recommendations in females and in the elderly [39]. Guidelines should be based not only on randomized studies but also on clinical experience as shown in real-world data.

Limitations and strengths: patients hospitalized for MDD were studied, so the results cannot be generalized to outpatients or to people with other illnesses. Due to the lack of a control group, the changes observed in prescription behavior after publication of the DDLs cannot necessarily be attributed to them. The prescription data of the AMSP project are cross-sectional data and do not allow any statement about the course of prescriptions for individual patients or their ADRs. Due to the inpatient setting, AMSP is able to measure actual utilization rates versus merely prescription rates as in most ambulatory settings. Further, the large sample size and the long observation period are major strengths of the present study.

## Conclusion

Although the instructions in the DDLs were inadequately followed, the actual prescribing practices are quite understandable. It appears that it was difficult for treating physicians to strictly adhere to all the suggestions made in the DDLs, e.g., not exceeding the maximum dosages of es-/citalopram and simultaneously avoiding combination treatments with other potentially QTc-prolonging drugs, especially in the elderly. Sertraline, which is more favorable regarding cardiac risks, was prescribed more to the detriment of citalopram. The trend towards prescribing fewer tranquillizing and hypnotic drugs continued. DDLs should give clear instructions that are easy to follow and should be better tailored to real clinical needs. Although this particular guideline is controversial, it is highly important that clinicians adhere closely to guidelines and are aware of the risks of high dosages and risky combinations.

## Supplementary Information

Below is the link to the electronic supplementary material.

Supplementary file1 (DOCX 98 KB)
