# Supplementary material for: Dear Doctor Letters regarding citalopram and escitalopram: guidelines vs real-world data
Source: Eur Arch Psychiatry Clin Neurosci. 2022 Feb 25;273(1):65–74. doi: 10.1007/s00406-022-01392-x (PMC9957836; doi:10.1007/s00406-022-01392-x)
Supplement: Supplementary file 1 — Supplementary file1 (DOCX 98 KB) [file 406_2022_1392_MOESM1_ESM.docx]

**Supplementary Material**

**Dear Doctor Letters regarding Citalopram and Escitalopram: Guidelines vs real world data**

Mateo de Bardeci*^1,2,^, Waldemar Greil*^1,2,^ Hans Stassen^2,3^, Jamila Willms^2^, Ursula Köberle^4^, René Bridler^2^, Gregor Hasler^5^, Siegfried Kasper^6^, Eckart Rüther^1^, Stefan Bleich^7^, Sermin Toto^7^, Renate Grohmann*^1^, Johanna Seifert*^7^

*equal contribution

^1^Department of Psychiatry and Psychotherapy, Ludwig Maximilian University, Munich, Germany

^2^Psychiatric Private Hospital, Sanatorium Kilchberg, Kilchberg-Zurich, Switzerland

^3^Institute for Response-Genetics, Psychiatric University Hospital (KPPP), Zurich, Switzerland

^4^Arzneimittelkommission der Deutschen Ärzteschaft, Berlin, Germany

^5^Psychiatry Research Unit, University of Fribourg, Switzerland

^6^Department of Molecular Neuroscience, Medical University of Vienna, Austria

^7^Department of Psychiatry, Social Psychiatry and Psychotherapy, Hannover Medical School, Hannover, Germany

Corresponding author:

Prof. Dr. Waldemar Greil , Psychiatric Department, Ludwig Maximilian University Munic

**Table 1 Suppl.**

Psychotropic drugs: Risk of QTc prolongation and / or of Torsade de pointes (TdP)

According to Wenzel-Seifert et al. 2011, Table in supplementary material

QTc prolongation TdP Risk

Arizona CERT

Thiorizadine +++ 1

Pimozide +++ 1

Methadone +++ 1

Levomethadone +++ 1

Chlorpromazine ++ 1

Haloperidol + 1

Sertindole +++ 2

Quetiapine +++ 2

Lithium +++ 2

Risperidone ++ 2

Clozapine ++ 2

Ziprasidone ++ 2

Venlafaxine + 2

Chloralhydrate + 2

Paliperidone ER - 2

Amitriptyline +++ 3

Doxepine +++ 3

Imipramine +++ 3

Desipramine +++ 3

Nortriptyline +++ 3

Fluoxetine ++ 3

Clomipramine ++ 3

Maprotiline +++ -

Melperone +++ -

Levomepromazine ++ -

Sulpiride ++ -

QTc prolongation

+ mild (>5 and <9 ms) or only in case of overdose or intoxication (not calculated as risky)

++ moderate (≥9 and <16ms)

+++ severe (≥17ms)

TdP risk according to the Arizona CERT

1. generally accepted elevated risk of TdP

2. rare cases of TdP, possible but not adequately documented TdP risk

3. weak association with TdP; (not calculated as TdP risk)

- no risk of TdP reported

All psychotropic substances with +++ or ++ in QTc prolongation and/or 1. and 2. in TdP risk were classified as risky drugs.

**Table 2 Suppl.** Study Population (Major Depressive Disorder (MDD), 2001 – 2017, all)

| Total number of patients | 43,480 | - | - |
| --- | --- | --- | --- |
| Number of males | 16,128 | - | 37.1% |
| Number of females | 27,352 | - | 62.9% |
| Average age males | 49.1 | SD: | 15.5 |
| Average age females | 51.6 | SD: | 16.6 |
| Mild MDD ("F32.0", "F33.0") | 497 | - | 1.1% |
| Males | 206 | - | 1.3% |
| Females | 291 | - | 1.1% |
| Moderate MDD ("F32.1", "F33.1”) | 11,437 | - | 26.3% |
| Males | 4,191 | - | 26.0% |
| Females | 7,246 | - | 26.5% |
| Severe MDD ("F32.2", "F33.2") | 24,912 | - | 57.3% |
| Males | 9,207 | - | 57.1% |
| Females | 15,705 | - | 57.4% |
| Severe MDD with psychosis ("F32.3", "F33.3") | 5,499 | - | 12.6% |
| Males | 2,096 | - | 13.0% |
| Females | 3,403 | - | 12.4% |
| Other or no info MDD (“F32”, “F32.8", “F32.9", “F33”, "F33.4", “F33.8“, "F33.9") | 1,129 | - | 2.6% |
| Males | 425 | - | 2.6% |
| Females | 704 | - | 2.6% |

SD: Standard deviation

The data for the study population was taken from the AMSP dataset with the following constrains: 2001-2017, age ≥ 18 and <90, diagnosis codes F32- and F33- and at least one prescription of a psychotropic drug.

**Figure 1 Suppl.** Percent of patients treated with the respective psychotropic drug group

**
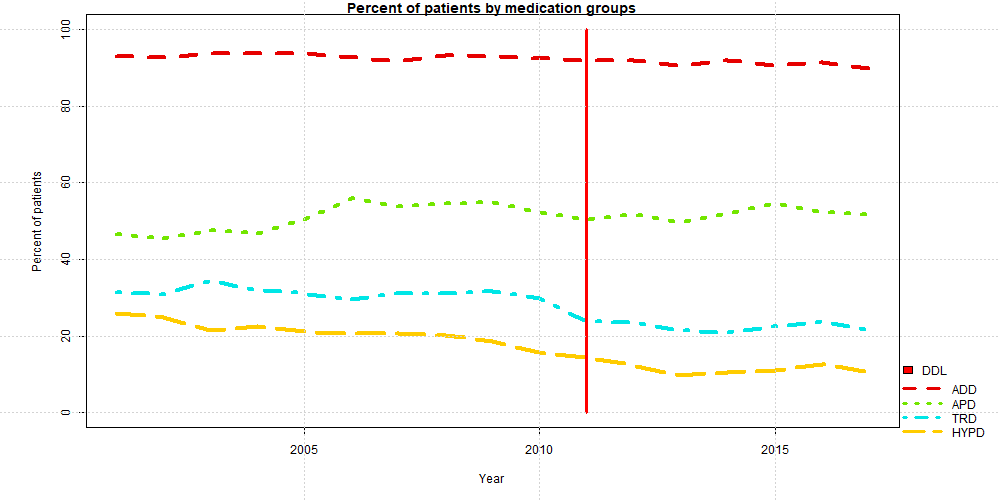
**

Prescription of classes of psychotropic drugs: Antidepressant drugs (ADD), Antipsychotic drugs (APD), Tranquilizing drugs (TRD), Hypnotic drugs (HYPD)

The trends of decreasing prescriptions of TRD and HYPD appear to be unaffected by Dear Doctor Letters (DDL) in 2011; no essential change for ADD and APD.

**Figure 2 Suppl.** Percent of patients treated with antidepressant drugs


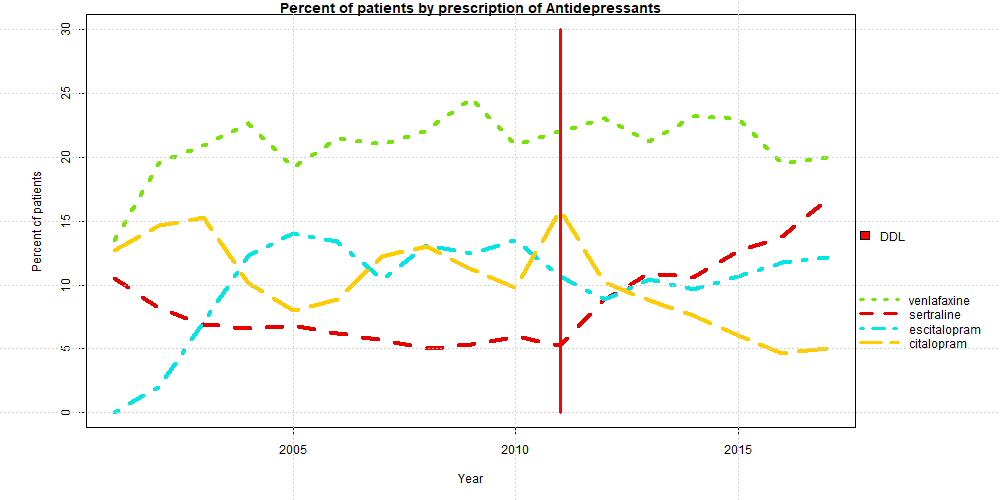


Prescription of citalopram, escitalopram, sertraline and venlafaxine

Within the group of ADD there was a decrease of prescriptions of citalopram while sertraline prescriptions increased after DDL in 2011.

**Figure 3 Suppl.** Combination of es-/citalopram with quetiapine

a)


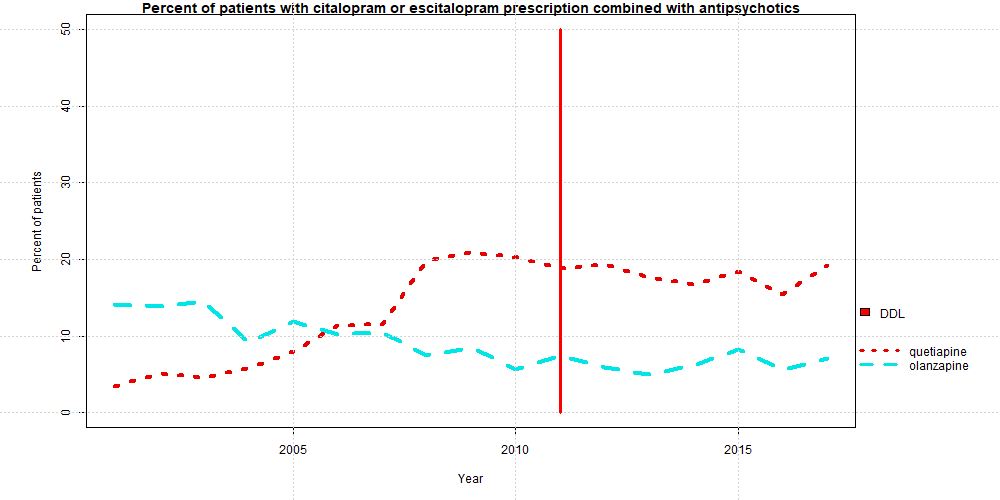


b)


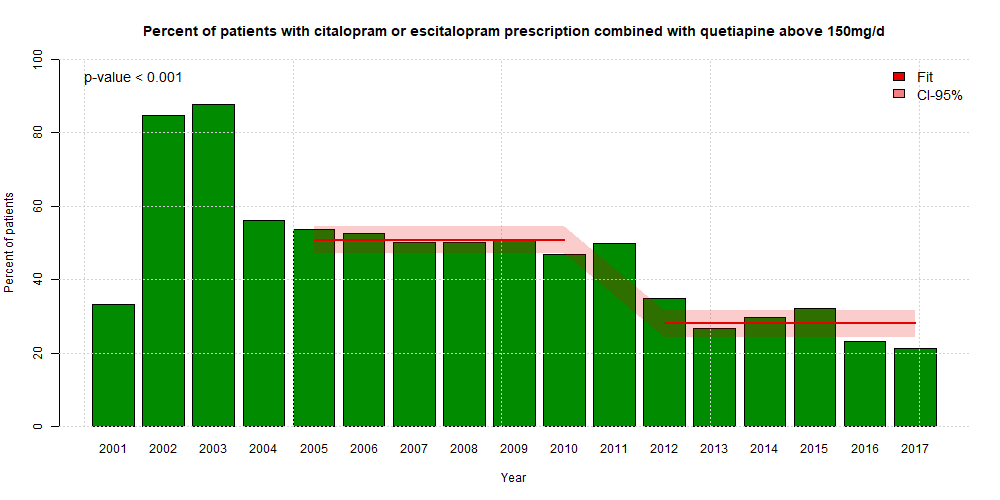


1. Percent of patients treated with es-/citalopram combined with quetiapine (n=1097) or olanzapine (n= 600), the two most prescribed combinations of es-/citalopram with antipsychotics.
2. Percent of patients dosed above 150mg/d of quetiapine in combination with es-/citalopram.

The percentage of combinations of es-/citalopram with quetiapine remained rather stable before and after DDL in 2011 (about 20%; Figure a), but the proportion of “high” dosages of quetiapine (>150mg/d) in combination with es-/citalopram decreased significantly after DDL in 2011 (p<.001; Figure b).

The proportion of es-/citalopram patients on quetiapine above 150 mg/d reduced from 51% to 28%; exact values: 50.7% (SE 1.7) to 27.9% (SE 2.3), p<.001, comparing six years before and after DDL. Three-year comparison values: 49% to 30%. On the other hand, the proportion of patients dosed 50mg quetiapine or lower increased from 22.3% to 41.8% (six years; three-year comparison 22.4% to 37.6%).

After the DDLs, an increasing proportion of es/citalopram patients in combination with es-/citalopram were treated with low doses of quetiapine (up to 50 mg/d), i.e., doses that are sedative and sleep inducing rather than antidepressant.

Note: The most prescribed antipsychotic drugs in combination with es-/citalopram in the study population were (number of patients, n ≥ 50): quetiapine (1097), olanzapine (600), risperidone (462), pipamperone (329), promethazine (313), prothipendyl (271), melperone (190), chlorprothixene (181), aripiprazole (164), amisulpride (91), perazine (63), haloperidol (50).

**Figure 4 Suppl.** Polypharmacy with psychotropic drugs


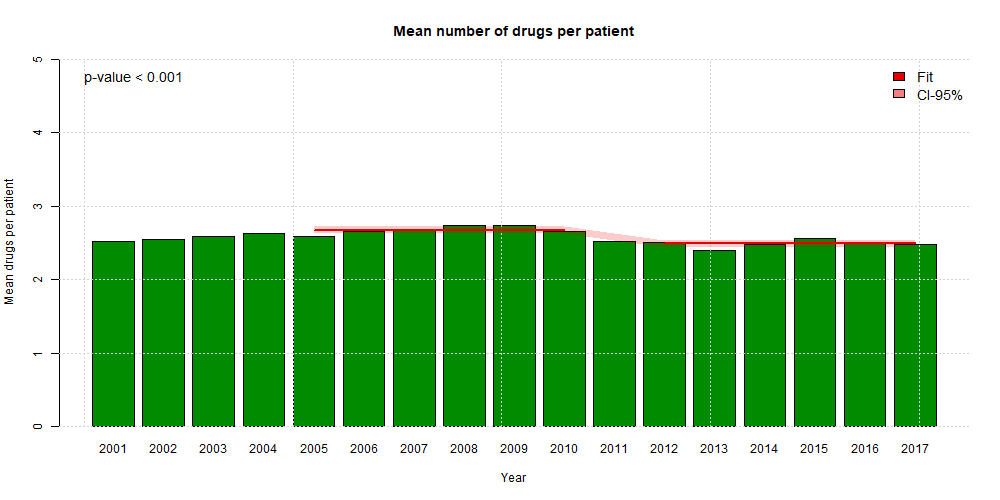


Mean number of prescribed psychotropic drug per patient: a slight decrease of psychotropic combinations before and after DDL in 2011, yielding a statistically significant result due to low variance (p<.001).

The number of concomitant psychotropic medications prescribed decreased from 2.7 (SE 0.02) in 2005-2010 to 2.5 (SE 0.03) in 2012-2017, p<.001, when comparing six years before and after the DDL. The three-year comparison showed almost identical results: 2008-2010: 2.7 (SE 0.03) to 2012-2014: 2.5 (SE 0.04)

**Figure 5 Suppl.** Citalopram combinations with antipsychotic drugs


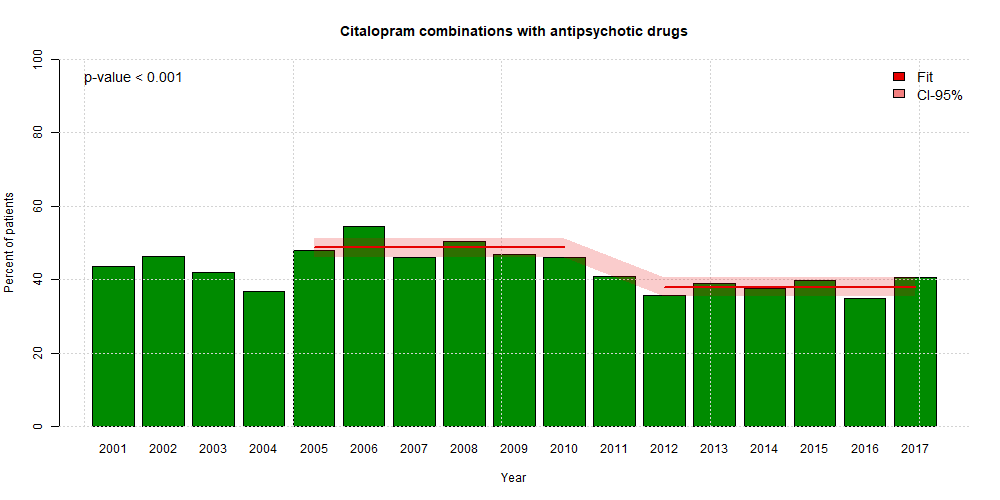


Combinations of escitalopram with APD (in percent)

Statistics 2005-2010 vs 2012-2017: 48.63% versus 37.9%, p < .001

(For comparison: 2008-2010 vs 2012-2014: 47.8% versus 37.4% %, p < .003)

**Figure 6 Suppl**. Escitalopram combinations with antipsychotic drugs
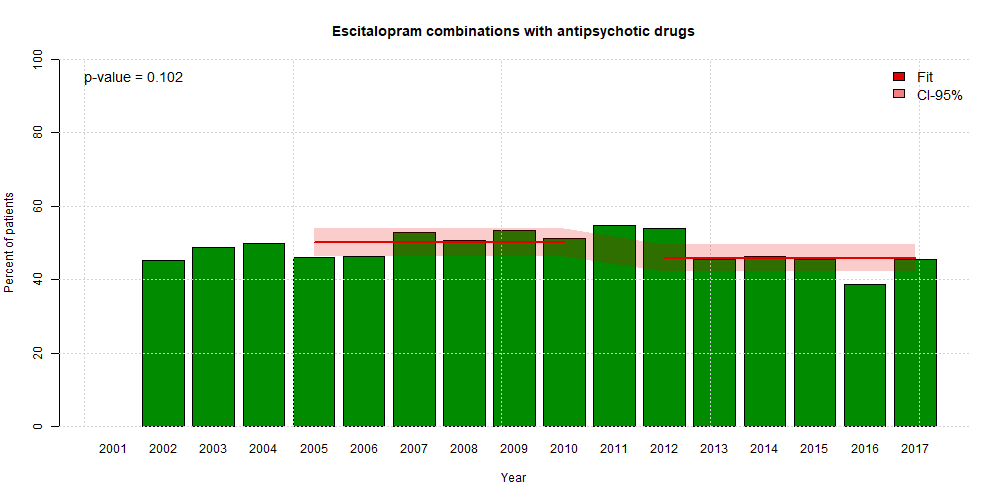


Combinations of escitalopram with APD (in percent)

Statistics 2005-2010 vs 2012-2017: 50.1% versus 45.8% (n.s.)

(For comparison: 2008-2010 vs 2012-2014: 51.8% versus 48.5% (n.s)
